# *Salmonella enterica* Serovar Panama, an Understudied Serovar Responsible for Extraintestinal Salmonellosis Worldwide

**DOI:** 10.1128/IAI.00273-19

**Published:** 2019-08-21

**Authors:** Caisey V. Pulford, Blanca M. Perez-Sepulveda, Ella V. Rodwell, François-Xavier Weill, Kate S. Baker, Jay C. D. Hinton

**Affiliations:** a Functional and Comparative Genomics, Institute of Integrative Biology, University of Liverpool, Liverpool, United Kingdom; b Institut Pasteur, Unité des Bactéries Pathogènes Entériques, Paris, France; Texas A&M University Health Science Center

**Keywords:** invasive nontyphoidal *Salmonella*, *Salmonella*, *Salmonella enterica* serovar Panama

## Abstract

In recent years nontyphoidal *Salmonella* has emerged as one of the pathogens most frequently isolated from the bloodstream in humans. Only a small group of *Salmonella* serovars cause this systemic infection, known as invasive nontyphoidal salmonellosis. Here, we present a focused minireview on Salmonella enterica serovar Panama, a serovar responsible for invasive salmonellosis worldwide. *S*. Panama has been linked with infection of extraintestinal sites in humans, causing septicemia, meningitis, and osteomyelitis. The clinical picture is often complicated by antimicrobial resistance and has been associated with a large repertoire of transmission vehicles, including human feces and breast milk. Nonhuman sources of *S*. Panama involve reptiles and environmental reservoirs, as well as food animals, such as pigs. The tendency of *S*. Panama to cause invasive disease may be linked to certain serovar-specific genetic factors.

## Abstract (precis)

In recent years nontyphoidal *Salmonella* has emerged as one of the pathogens most frequently isolated from the bloodstream in humans. Only a small group of *Salmonella* serovars cause this systemic infection, known as invasive nontyphoidal salmonellosis. Here, we present a focused minireview on Salmonella enterica serovar Panama, a serovar responsible for invasive salmonellosis worldwide. *S*.

## INTRODUCTION

Salmonellosis is a disease caused by the enteric pathogen Salmonella enterica, a species that includes 2,637 different serovars (1). The various clinical presentations of *Salmonella* disease in humans include enteric fever, gastroenteritis, extraintestinal complications, and a chronic carrier state (2, 3). The clinical manifestation of *Salmonella* is dependent on a number of features, including host immune status (reviewed in reference 4), as well as factors specific to the *Salmonella* pathovariant that is causing the infection (5). Certain pathogen factors are associated with clinical presentation, including serovar and certain core and accessory genome components, such as the presence of plasmids, prophages, virulence factors, and antimicrobial resistance genes (6). In this review, we focus on Salmonella enterica serovar Panama, which has a strong association with invasive disease (7) and is a rarely discussed serovar that has global public health relevance. We review the global epidemiology, as well as the clinical picture, the transmission vehicles, and antimicrobial resistance, and put them into the context of our current genomic understanding.

## GLOBAL DISEASE BURDEN AND EPIDEMIOLOGY

In 1931, an unknown bacterium caused widespread foodborne diarrheal disease among American soldiers stationed at the Panama Canal. A full microbiological investigation was conducted, and the organism was identified as a “not previously described *Salmonella*,” which was subsequently named *S*. Panama (8). Since initial isolation and serological characterization, *S*. Panama has been implicated in numerous geographically localized outbreaks of gastrointestinal and extraintestinal disease around the globe (9).

### French territories in the Americas.

*S*. Panama is responsible for a significant proportion of the total *Salmonella* disease burden worldwide and is a leading cause of invasive nontyphoidal salmonellosis in French territories of America located in the Caribbean and South America (7, 10, 11). Between 1972 and 1974, *S*. Panama was the major *Salmonella* serovar isolated from human fecal samples in Martinique (10). Two decades later, a study focused on pediatric salmonellosis in Martinique identified *S*. Panama as the most commonly isolated *Salmonella* serovar, accounting for 35% of all cases between 1990 and 1994 (11). Similarly, in French Guiana, *S*. Panama was the most frequent *Salmonella* serovar acquired by humans, accounting for 12.9% of all cases of *Salmonella* infection in 2011 (12). More recently, *S*. Panama was listed as the *Salmonella* serovar most frequently isolated from pediatric blood samples in Guadeloupe, contributing to one-third of all cases of *Salmonella* infection between 2010 and 2014 (7), and univariate analysis showed *S*. Panama was associated with causing disease in children older than 6 months of age (*P* = 0.002) (7). These examples demonstrate the significant impact that *S*. Panama has on public health in French territories in the Americas and shows that *S*. Panama causes extraintestinal infection and gastrointestinal disease, particularly in children. Although more extensive work needs to be done, no evidence for antimicrobial resistance in *S*. Panama exists in these regions.

### Latin America.

*S*. Panama causes a significant proportion of the salmonellosis burden in Latin America, which in the 2000s was 3.5 cases confirmed by serotyping per 100,000 people (9). As early as the 1950s, 41 (12%) of 357 human *Salmonella* isolates collected in Maracaibo, Venezuela, were *Salmonella* serovar Panama. Interestingly, 15 isolates came from patients suffering from gastroenteritis, 4 came from individuals with enteric fever, and 22 came from healthy carriers, indicating that *S*. Panama could be carried asymptomatically (13).

Historically, an outbreak of *S*. Panama in Chile originated from river water in Santiago in 1975 (14). By 1978, the serovar had infiltrated almost the entire country, expanding southward to Punta Arenas and northward toward Arica. The resulting human epidemic across Chile lasted for 4 years and involved the isolation of *S*. Panama from food, animals, and water, demonstrating the ability of the serovar to spread rapidly and survive outside of the human host. The majority of clinical cases involved children under 15 months of age with self-limiting diarrheal disease. However, examples of bacteremia and meningitis were also reported (14).

*S*. Panama continues to be isolated periodically in Chile and other parts of Latin America. According to global *Salmonella* monitoring compiled by the World Health Organization between 2001 and 2007, *S*. Panama was the ninth most common serovar isolated in Latin America (9). In 2007, *S*. Panama was responsible for 1% of 3,439 cases of *Salmonella* infection across Argentina, Brazil, Chile, and Costa Rica (9). In Colombia, *S*. Panama was the fifth most common serovar isolated from patients between 2005 and 2011 (15). Rapid dissemination of *S*. Panama around Chile in the 1970s, and the consistent reporting of the serovar among the top 10 that cause human disease post-2000, highlight the persistent burden of *S*. Panama in Latin America.

### Asia.

In Asia, *S*. Panama was the 11th most frequently isolated *Salmonella* serovar in humans between 2001 and 2007 (9). In 2001, 4% of salmonellosis cases in Thailand were caused by *S*. Panama, dropping to 3% in 2007 (9). In Tokyo, Japan, *S*. Panama was the third most common *Salmonella* serovar between 1974 and 1979, accounting for 5% of cases of *Salmonella* infection, and was commonly isolated from asymptomatic people (16). In Taiwan, where *S*. Panama causes 7% of the clinical cases of salmonellosis, *S*. Panama causes a higher rate of bacteremia in children under 5 years of age than other serovars, such as Salmonella enterica serovar Enteritidis (17). These findings demonstrate that *S*. Panama is an important public health issue in Asia.

### Europe and the United States of America.

Historically, *S*. Panama has caused a significant proportion of the salmonellosis cases in Europe, particularly related to the pig industry, and in the United States, where *S*. Panama has been implicated in several hospital and statewide outbreaks associated with a variety of food sources (18, 30, 98). The serovar was introduced into the United Kingdom during World War II as a result of unsterilized dried eggs imported from the United States being fed to pigs (18). Humans have also been involved in the spread of *S*. Panama during hospital outbreaks in France and in other Western European countries during the 1960s and 1970s (19, 20). Over this period, there was a 3-fold increase in *S*. Panama cases in the United Kingdom, which led to a doubling of the number of salmonellosis cases (18). Subsequently, between 1969 and 1984, *S*. Panama was one of the top five serovars responsible for invasive disease in the United Kingdom (21). It is thought that these isolates were exposed to high antibiotic selective pressure in humans or food animals and consequently became resistant to antibiotics via acquisition of many types of plasmids (22–28). Elsewhere in the European Union, *S*. Panama was reported among the top 10 most frequently isolated serovars during 2012, following 706 confirmed cases of *S*. Panama salmonellosis associated with outbreaks in Germany and Italy (29). Sporadic outbreaks of *S*. Panama salmonellosis also occurred in Switzerland (1972), Hungary (1979), Spain (1998), and the Netherlands (2008) (30–33). *S*. Panama maintained its ranking in the top 20 serovars associated with salmonellosis in the European Union until 2017, when it was replaced by other serovars (Salmonella enterica serovar Brandenburg, Salmonella enterica serovar Kottbus, and Salmonella enterica serovar Coeln) (34).

## CLINICAL PICTURE IN HUMANS

**FIG 1:**
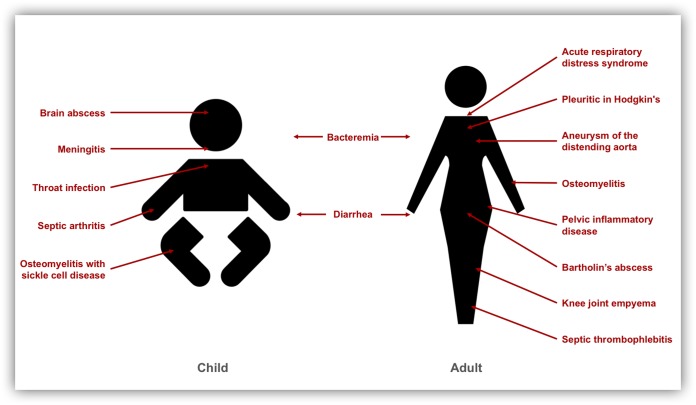
Overview of the clinical presentations caused by *S*. Panama in adults and children according to the published literature, as follows: baby, brain abscess (36), meningitis (8, 14, 36, 38–43, 45), throat infection (35), septic arthritis (91), and osteomyelitis with sickle cell disease (92); adult, acute respiratory distress syndrome (93), pleuritic in Hodgkin’s disease (94), aneurysm of the distending aorta (95), osteomyelitis (40, 92), pelvic inflammatory disease (96), Bartholin’s abscess (37), knee joint empyema (40), and septic thrombophlebitis (97).

Although *S*. Panama can cause gastrointestinal infection in humans (9), the serovar is more widely known for its ability to cause invasive disease and to colonize extraintestinal sites. For most salmonellae, extraintestinal colonization refers to bloodstream infection (2). However, *S*. Panama can also invade specific body sites, causing atypical presentations, including throat infection, brain abscess, and Bartholin’s abscess (35–37) (summarized in Fig. 1). These unexpected symptoms of *S*. Panama infection can impede diagnosis and delay treatment.

The clinical presentation of *S*. Panama disease varies between adults and children (Fig. 1). A common complication of neonatal *S*. Panama infection is the development of *Salmonella* meningitis (8, 14, 36, 38–45), a lethal disease that has previously been linked to localized outbreaks in hospital maternity wards (8, 31). For example, *S*. Panama was recovered from 138 babies, new mothers, and staff during an outbreak of salmonellosis in a neonatal nursery in Michigan in 1934 to 1944 that resulted in 18 fatalities due to *Salmonella* meningitis (8). Similar outbreaks have historically occurred in other countries, including Germany, where a hospital outbreak in a maternity unit caused prolonged contamination despite radical disinfection of the entire ward (46).

*S*. Panama causes more cases of clinically invasive disease in humans than most *Salmonella* serovars. Historically, *S*. Panama infections have been 11 times more likely to cause invasive disease than those by other serovars in Martinique (10, 11). In England, 7% of all *S*. Panama isolates were isolated from extraintestinal sites compared to 2% of Salmonella enterica serovar Typhimurium and 3% of *S*. Enteritidis isolates (21). In Taiwan, 70% of *S*. Panama isolates were isolated from invasive disease compared to 12% of *S*. Enteritidis isolates (47). In addition to these epidemiologically suggestive data, multivariate analysis has recently confirmed the association of *S*. Panama with clinically invasive infection (*P* < 0.001) as part of a retrospective study of *Salmonella* infections in children living in Guadeloupe (7). A gnotobiotic-mouse model has been described for *S*. Panama (48), which could help to elucidate the mechanisms behind the increased invasiveness.

## TRANSMISSION VEHICLES

Wild reptiles are the natural reservoir for *S*. Panama in Latin America (12, 49–52). A study focusing on the frequency and host distribution of *Salmonella* serovars in reptiles and amphibians captured in the Republic of Panama between 1965 and 1967 showed that 2.6% of 78 *Salmonella* isolates were serovar Panama (49). In a subsequent study (1966 to 1969), 6.8% of *Salmonella* organisms isolated from neotropical lizards in Panama were *S*. Panama (50). In the past decade, a high prevalence of *Salmonella* has been found in the largest lizards in South America (Tegu lizards), and 3% of the isolates were classified as *S*. Panama (51). In French Guiana, where *S*. Panama was the most frequently isolated human-associated serovar in 2011, the serovar was also isolated from wild reptiles (12). Reptiles are likely to be an important source for transmission of *S*. Panama in regions of the world where many lizards and other reptiles are present in and around households. A recent survey of *Salmonella* strains carried by African venomous snakes did not isolate *S*. Panama (53).

In addition to reptiles, *S*. Panama has also been isolated from other wildlife species and companion animals. A study on pouched wild birds found *S*. Panama in cloacal swabs of chestnut-capped blackbirds in Rio de Janeiro, Brazil (54). In regard to companion animals, *S*. Panama was isolated from a household dog in Taiwan (55). *S*. Panama contamination has been found in birds and fish tanks sampled from pet shops and households in Trinidad (56). Wildlife, therefore, represent a potential reservoir for *S*. Panama dissemination.

In Europe, *S*. Panama infection is primarily a foodborne disease, with the main transmission vehicles being pork-derived products, including cured meat, minced pork, and sausages (57). The transmission pathway for *S*. Panama begins in animal feed, from where it can enter porcine and poultry animal reservoirs and move into animal food products, eventually infecting humans (18).

At the animal level, *S*. Panama was found in 2.08% of 200 abattoir pigs sampled in Budapest, Hungary (58), and has been found in cattle and swine in Germany (59). Outside Europe, *S*. Panama has been identified in beef and dairy herds in Argentina (60) and is the second most common *Salmonella* serovar to be isolated from swine finishing herds in Brazil (61).

*S*. Panama is also recognized as a contaminant in food-processing facilities and retail establishments globally, including butcher shops (62), public markets (63), meat vans (64), and slaughterhouses (65). The process of manufacturing pork-derived products includes several steps designed to result in a microbiologically safe, shelf-stable product by tightly controlling physicochemical conditions, such as salt and nitrate concentrations, pH, water activity, and temperature (66). However, *Salmonella* viability throughout this curing process has been reported, including the presence of *S*. Panama in salami (67, 68). In the Netherlands, *S*. Panama has additionally been implicated in the contamination of cattle-derived food products and was one of the three *Salmonella* serovars most frequently isolated from mincemeat over a 13-month period. Interestingly, mincemeat from slaughterhouses was more likely to contain *Salmonella* than mincemeat derived from slaughtering completed at butcher shops (69). Food-processing facilities themselves can play a role in the contamination of animal food products with *S*. Panama.

The impact of *S*. Panama entering the human food chain can be seen in an outbreak of salmonellosis that affected 300 people who had eaten contaminated roast pork in the United Kingdom in 1970. *S*. Panama was implicated as the etiological agent (18). *S*. Panama has also caused several foodborne outbreaks between 1990 and 1999 in Asturias, Spain, and isolates were collected from gastroenteritis and septicemia patients who had consumed contaminated fish puddings, cooked octopus, and cream cakes (32). Other studies have linked *S*. Panama infections to consumption of goat cheese, vegetables, beef, poultry, eggs, fruit juice, and shellfish (14, 33, 70).

In addition to the usual fecal-oral transmission route of *Salmonella* in humans, breast milk has also been suggested as a vector for *S*. Panama (71). A study demonstrated that *S*. Panama can infect the human mammary duct, can be shed for at least 2 weeks, and can remain stable during storage of breast milk at 4°C (71). Furthermore, it is possible that a case of meningitis in an exclusively breastfed 4-month-old patient was contracted from breast milk that was contaminated with an antimicrobial-susceptible *S*. Panama isolate (41).

## ANTIMICROBIAL RESISTANCE

### Burden of antimicrobial resistance in *S*. Panama.

Antimicrobial resistance (AMR) is an important public health concern (72). There are conflicting reports in the literature relating to the AMR status of the *S*. Panama serovar, with studies in Italy and Brazil reporting low levels of antibiotic resistance (41, 73). They are supported by further reports from Martinique, where 91% of *S*. Panama isolates were susceptible to beta-lactams (11), and Guadeloupe, where all *Salmonella* serovars demonstrated high overall susceptibility to antibiotics (7). In contrast, other studies have seen higher levels of resistance in *S*. Panama, particularly against tetracycline (e.g., 67%) and chloramphenicol (e.g., 67%) since the 1980s (24, 47, 59, 74–76). Antibiotic stewardship promises to be an effective tool for decreasing antimicrobial resistance in the *S*. Panama serovar. For example, following a ban on tetracycline use in the pork industry in the Netherlands, *S*. Panama tetracycline resistance dropped from 90% to 1% (24).

In Asia, *S*. Panama has been associated with high levels of AMR since 1980, when 58% of the *S*. Panama isolates from Tokyo were resistant to at least one antibiotic agent (77). This figure appears to be on the rise. By the turn of the millennium, 83% of domestic and imported *S*. Panama isolates from cases in Tokyo were multidrug resistant. Similarly, in Taiwan, the serovar also exhibited resistance to multiple antibiotics, including cotrimoxazole (67%), ampicillin (56%), streptomycin (56%), kanamycin (56%), and gentamicin (45%) (74). The high proportion of *S*. Panama isolates that show AMR should be considered by clinicians working in Asia and by health care practitioners globally when treating Asian-travel-associated salmonellosis cases caused by *S*. Panama.

### Genomic markers and trends in antimicrobial resistance.

A large proportion of *S*. Panama antimicrobial resistance has been associated with plasmid carriage (*P* = 0.012), class 1 integron presence, and transmissible drug resistance (R) factors (22, 47, 74, 78). Resistance to tetracycline, for example, has often been mediated by the R factor R1 in *S*. Panama (26). Such R factors have been implicated in the transfer of multiple antimicrobial resistance genes, usually simultaneously, between *S*. Panama strains and other bacteria. However, an isolate from an epidemic of *S*. Panama infection in Paris showed unusual patterns of transferable resistance, which may extend to other strains in the *S*. Panama serovar. The isolate was able to transfer genes involved in antimicrobial resistance singly or in pairs, rather than as one antibiotic resistance cassette. The proposed mechanism involved the simultaneous transfer of several discrete genetic elements that were able to coexist stably and to replicate noncompetitively in *S*. Panama. The authors suggested that frequent cotransfer of genetic elements may be propagated by conjugative-transfer machinery (27).

## INVASIVE DISEASE—GENOMIC INFERENCES IN *S*. PANAMA

### Evolutionary history and virulence.

The study of evolutionary history may explain why *S*. Panama is associated with invasive disease. The majority of salmonellae that cause disease in humans belong to S. enterica subsp. enterica, which is further divided into two main clades, A and B, and a number of smaller clades (79). Phylogenetically, *S*. Panama is in clade B, which is associated with increased levels of clinically invasive disease (53, 80, 81). Another review of the population structure within S. enterica found that *S*. Panama is in lineage 3 (equivalent to the above-mentioned clade B) (82).

The evolutionary history of *S*. Panama was studied by Selander et al. (83), who used multilocus enzyme electrophoresis to assess the relationships among *Salmonella* serovars that cause invasive disease. It was proposed that *S*. Panama evolved from the same ancestors that gave rise to Salmonella enterica serovar Paratyphi, Salmonella enterica serovar Sendai (which causes enteric fever), and Salmonella enterica serovar Miami (83). In the current era of genomically informed epidemiological analysis, phylogenetic methods can be used to understand the evolutionary history of *Salmonella*. However, no large-scale phylogenetic study has yet been conducted on *S*. Panama, and only one complete *S*. Panama genome sequence (from strain ATCC 7378; GenBank accession no. CP012346) is available (84). As part of the current review, virulence genes were identified in the complete genome of *S*. Panama strain ATCC 7378 using the program ABRicate v0.8.10 (https://github.com/tseemann/abricate) against a virulence factor database (85) with default parameters. In total, 131 virulence-associated genes were identified. The analysis confirmed the presence of typical *Salmonella* virulence determinants, including type III secretion systems, type III effector proteins, fimbriae, and flagella. Of interest, *S*. Panama was also found to carry the cytolethal distending toxin B gene (*cdtB*), which is characteristic of S. enterica clade B and the highly invasive Salmonella enterica serovar Typhi (53, 80, 81). A more detailed, epidemiologically representative analysis is required to further elucidate the uniqueness of the *S*. Panama serovar.

### Accessory genome and virulence.

Generally, plasmids play a key role in systemic *Salmonella* infection, but little is known about the plasmid complement of the *S*. Panama serovar. In the small number of available studies, it is reported that *S*. Panama, including the above-mentioned *S*. Panama ATCC 7378, does not commonly carry the large plasmids that have previously been associated with virulence in other *Salmonella* serovars (41). Rather, *S*. Panama strains carry a heterogeneous population of plasmids (86). Prophages can also make significant contributions to *Salmonella* virulence (87, 88), but only one study has reported the presence of prophages in *S*. Panama (84). The *Salmonella* RE-2010 prophage was identified in the genome of *S*. Panama ATCC 7378. The prophage (also known as ElPhiS) has also been found in *S*. Enteritidis, where it has been associated with specific phylogenetic clusters (89, 90). The importance of *S*. Panama for public health globally necessitates that a concerted comparative genomic analysis be conducted in the future.

## PERSPECTIVES

*S*. Panama is a globally relevant pathogen that has consistently been reported as one of the most frequently isolated *Salmonella* serovars over the past 70 years. The proportion of clinical cases caused by *S*. Panama is particularly high in French territories in the Americas, where it is associated with invasion of extraintestinal sites, particularly in infants. Reptiles act as natural reservoirs for *Salmonella* in these regions, and it has been speculated that the large numbers of reptiles found in and around homes in tropical regions of America lead to high levels of *S*. Panama transmission to humans. The serovar was also introduced into Europe, where it spread through the pork industry and caused hospital outbreaks in the 1960s and 1970s. *S*. Panama continues to contribute to the global disease burden caused by salmonellae.

It is important to highlight the unusual clinical presentation of *S*. Panama in different patient populations to avoid delays in patient treatment. Clinicians and researchers should remain aware of the potential for increasing levels of antimicrobial resistance in the serovar, as has been described in Asia. Unraveling the molecular epidemiology and evolutionary history of *S*. Panama is the obvious next step in understanding more about this rarely studied serovar that continues to cause invasive salmonellosis worldwide.
